# Early evaluation of sunitinib for the treatment of advanced gastroenteropancreatic neuroendocrine neoplasms via CT imaging: RECIST 1.1 or Choi Criteria?

**DOI:** 10.1186/s12885-017-3150-7

**Published:** 2017-02-23

**Authors:** Yanji Luo, Jie Chen, Kun Huang, Yuan Lin, Minhu Chen, Ling Xu, Zi-Ping Li, Shi-Ting Feng

**Affiliations:** 1 Department of Radiology, The First Affiliated Hospital, Sun Yat-Sen University, 58th, The Second Zhongshan Road, Guangzhou, Guangdong 510080 China; 2 Department of Gastroenterology, The First Affiliated Hospital, Sun Yat-Sen University, 58th, The Second Zhongshan Road, Guangzhou, Guangdong 510080 China; 3 Department of Pathology, The First Affiliated Hospital, Sun Yat-Sen University, 58th, The Second Zhongshan Road, Guangzhou, Guangdong 510080 China; 4 Faculty of Medicine and Dentistry, University of Western Australia, Perth, Australia

**Keywords:** Gastroenteropancreatic neuroendocrine neoplasms, Sunitinib, Time to tumor progression, Computed tomography

## Abstract

**Background:**

The aim of this study was to assess and compare the Response Evaluation Criteria in Solid Tumors version 1.1 (RECIST 1.1) and the Choi criteria in evaluating the early response of advanced gastroenteropancreatic neuroendocrine neoplasms (GEP-NENs) treated with sunitinib.

**Methods:**

Eighteen patients with pathologically proven advanced GEP-NENs treated with sunitinib were enrolled in the study. Pre- and post-treatment CT scans (plain, biphasic enhanced CT scan) were performed on all patients. Changes in the target tumor size and density from pre-treatment to 1.4–3.1 months after treatment were measured and recorded for each patient. Tumor responses were identified using RECIST 1.1 and Choi criteria. The time to tumor progression (TTP) for each patient was measured and compared between groups using the Kaplan-Meier method.

**Results:**

Among the 18 patients, 4 (22%) exhibited a partial response (PR), 9 (50%) exhibited stable disease (SD), and 5 (28%) experienced progressive disease (PD), using RECIST 1.1. However, based on the Choi criteria, 8 (44%) patients exhibited a PR, 4 (22%) exhibited SD, and 6 (33%) experienced PD. According to RECIST 1.1, the median TTP of PR, SD and PD group were 16.6, 10.8 and 2.3 months, respectively. The TTP of the PR group was significantly longer than that of the PD group (*P* = 0.007) but insignificant when compared to the SD group (*P* = 0.131). According to Choi criteria, the median TTP of PR, SD and PD group were not reached, 10.8 and 2.3 months, respectively. The TTP of the PR group was significantly longer than that of the SD (*P* = 0.026) and PD groups (*P* < 0.001).

**Conclusion:**

The Choi criteria appear to be more sensitive and more precise than RECIST 1.1 in assessing the early response of advanced GEP-NENs treated with sunitinib.

## Background

Neuroendocrine neoplasms (NENs) comprise a wide range of malignancies originating from the neuroendocrine cells throughout the human body that constitute the endocrine system, or they may be derived from the diffuse neuroendocrine system [1]. The incidence of NENs in the last 30 years has significantly increased, from an estimated incidence of 1.09/10^5^ in 1973 to 5.05/10^5^ in 2004 [2]. Gastroenteropancreatic (GEP)-NENs are the most common type of NENs, accounting for 67.5% of all NEN cases [3]. These tumors are categorized as functional or nonfunctional based on the presence of hormone production, biological effects, and symptoms. Approximately 20% of GEP-NENs have been estimated to be functional [1–3]. Furthermore, WHO 2010 classifications distinguish GEP-NENs into well-differentiated and poorly differentiated neoplasms. Well-differentiated GEP-NENs are considered to be neuroendocrine tumors and are graded as G1 (mitotic count <2 per 10 high power fields (HPFs) and/or Ki67 ≤ 2%) or G2 (mitotic count 2–20 per 10 HPFs and/or Ki67 3–20%). Poorly differentiated GEP-NENs are considered to be neuroendocrine carcinomas and are graded as G3 (mitotic count >20 per 10 HPFs and/or Ki67 > 20%) or mixed adenoneuroendocrine carcinomas [4].

Surgical resection alone can be curative in patients with early-stage diseases [5, 6]. Unfortunately, more than half of patients are diagnosed with advanced disease on initial presentation which are not amenable to curative resection alone at the time of diagnosis [1, 7]. Streptozocin, used either alone or in combination with doxorubicin and/or 5-fluorouracil, remains the only cytotoxic chemotherapeutic agent approved for the treatment of advanced GEP-NENs [8, 9]. However, only variable and unsustainable outcomes have been observed, and its high toxicity profile further limits its clinical use [10]. For functional tumors, somatostatin analogues have been widely used for symptomatic relief, but they have limited antitumor activity [11, 12]. Newly developed targeted treatments, such as sunitinib malate (SUTENT; Pfizer Inc., New York, NY, USA), which potently inhibits a number of receptor tyrosine kinases, including vascular endothelial growth factor receptors (VEGFRs) 2 and 3, platelet-derived growth factor receptors (PDGFRs) α and β and the stem-cell factor receptor (c-kit) [13, 14], appear to be effective in the treatment of GEP-NENs. It is believed that tissues from these tumors exhibit widespread expression of these receptors, and inhibition by sunitinib blocks signal transduction, thereby reducing tumor growth, progression and metastasis [15, 16]. Sunitinib malate has shown clear clinical benefits for advanced GEP-NENs in phase II and III trials [17–19]. Evaluating tumor response is an arduous task due to the increasing use of sunitinib in the treatment of GEP-NENs. Response Evaluation Criteria in Solid Tumors (RECIST) is a well-established tool for the assessment of tumor response in clinical trials and one of the most commonly used sets of criteria that only considers long-term changes as its parameters [20]. However, recent studies have suggested that the introduction of targeted therapies may have no major effect on tumor size, despite reducing tumor vascularization and, consequently, tumor density [21, 22]. Thus, RECIST may significantly underestimate the tumor response to targeted therapies [23]. The ongoing challenge of evaluating the tumor response to targeted therapies prompted Choi et al. to develop composite criteria that integrate changes in both tumor size and density to evaluate the tumor response to imatinib, another targeted agent used in gastrointestinal stromal tumors (GISTs). The Choi criteria appeared to be more accurate in predicting drug efficacy than RECIST for GISTs treated with imatinib [24]. Similar findings have been observed in other solid tumors, such as hepatocellular carcinoma (HCC) and renal cell carcinoma [25–28]. Several diagnostic techniques have been widely used for monitoring the course of treatment and surveillance of GEP-NENs, including high-frequency or contrast-enhanced ultrasound echography, dynamic contrast-enhanced magnetic resonance imaging, and 18-fluorodeoxyglucose positron emission tomography scanning. The main drawbacks of these investigations include their cost, reproducibility, inter-observer variability, and limited availability [24]. Due to its panoramic capabilities and high spatial resolution, only contrast-enhanced computed tomography (CT) can be considered a reliable method for assessing both tumor size and tissue density [24].

Faivre S,and Dreyer C have suggested that Choi criteria might be considered as an alternative to RECIST to evaluate the effects of sunitinib in patients with advanced pancreatic neuroendocrine tumors with a small sample size (*n* = 10) did not enrolled the midgut NENs [25, 26]. In this study, we aimed to assess whether the Choi criteria could be used as a tool for quantitatively evaluating tumor response as an alternative to RECIST in advanced GEP-NENs treated with sunitinib.

## Methods

### Patients and clinical follow-up

In this retrospective study, patients with pathologically confirmed advanced GEP-NENs treated with sunitinib in our institution between January 2010 and October 2015 were selected. The trial was approved by the Institutional Review Board of Sun Yat-Sen University, and all patients enrolled in this study provided written informed consent for the research study protocol. All methods were carried out in accordance with the approved guidelines. Additional inclusion criteria involved the following: a minimal cumulative duration of 4 weeks of sunitinib treatment in patients with adequate hematologic, hepatic, and renal function. Patients underwent baseline thoracic, abdominal and pelvic CT scans within 3 weeks before sunitinib administration and an early evaluation of the tumor with a second CT scan within 1.4–3.1 months after the initiation of sunitinib treatment and every 2–3 months thereafter. Patients with non-evaluable lesions (largest diameter of the target lesion smaller than 1.0 cm) or whose scans were performed outside of the predefined interval were excluded. Patients with missing data due to poor follow-up compliance or premature death before the early evaluation were also excluded.

### Treatment

Patients received a continuous daily administration of sunitinib at an initial dose of 37.5 mg and were followed-up with on a monthly basis to assess clinical response and tolerance. A reduction in the dose to 25 mg was permitted in patients experiencing severe adverse events. Treatment was continued until confirmed disease progression was documented, unacceptable adverse events were observed, or premature death has occurred. Tumor progression was identified on the basis of the following CT findings: the appearance of new lesions or metastasis, the appearance of new intratumoral nodules, or an increase in overall tumor size of greater than 20% [20].

### Imaging techniques

All patients received enemas the night before their CT scans and fasted for a minimum of 6–8 h prior to the scans. All patients were given 1.6–2.0 L of 2.5% mannitol one hour before and 0.4–0.5 L at 45, 30, and 15 min before CT to ensure adequate bowel distension. The rectum was also distended using a 2.5% mannitol enema. Scans of the chest, abdomen, and pelvis were performed using a multi-slice CT scanner (Aquilion64, Toshiba Medical Systems, Tokyo, Japan) with the following scan parameters: tube voltage – 120 kV; tube current – 200 mA; beam collimation – 6 × 0.5 mm; slice thickness – 0.5 mm; slice increments – 0.5 mm; and pitch – 0.9. After the non-contrast scan, iodinated contrast (Ultravist 300, Bayer Schering, Berlin, Germany) at a concentration of 300 mg iodine/mL was administered at a flow rate of 3–4 ml/s via a needle cannula placed in the antecubital vein using an automatic injector with a volume of 1.5 mL/kg, followed by a 40-mL bolus of saline solution. After unenhanced scanning, arterial and portal venous phase acquisitions were obtained at 35 s and 65 s after the initiation of contrast medium injection, respectively. The unenhanced and portal venous phase scanning were performed on the chest, abdomen and pelvis, and the arterial phase scanning was only performed on the abdomen.

### Imaging analysis

At the end of the study, a radiologist with 14 years of experience in abdominal imaging who was blinded to the clinical data reviewed the baseline and all follow-up CT images independently in a randomized order. The CT images were analyzed according to the following parameters: target lesion detection, target lesion size (in centimeters) and density in Hounsfield Units (HU), and the TTP.

#### Target lesions

In the baseline CT images, the target lesion was required to be ≥1.0 cm in the largest dimension according to the selection criteria. Malignant thrombosis, malignant ascites or pleural effusion (confirmed by cytological examination of the fluid), and a lymph node with a short diameter ≤1.5 cm were considered to represent non-target lesions. A maximum of two lesions per organ and five total lesions per patient were selected according to RECIST version 1.1 recommendations [20]. For the Choi criteria, the same target lesions selected in RECIST version 1.1 were used [23, 24].

#### Tumor size

**Fig. 1:**
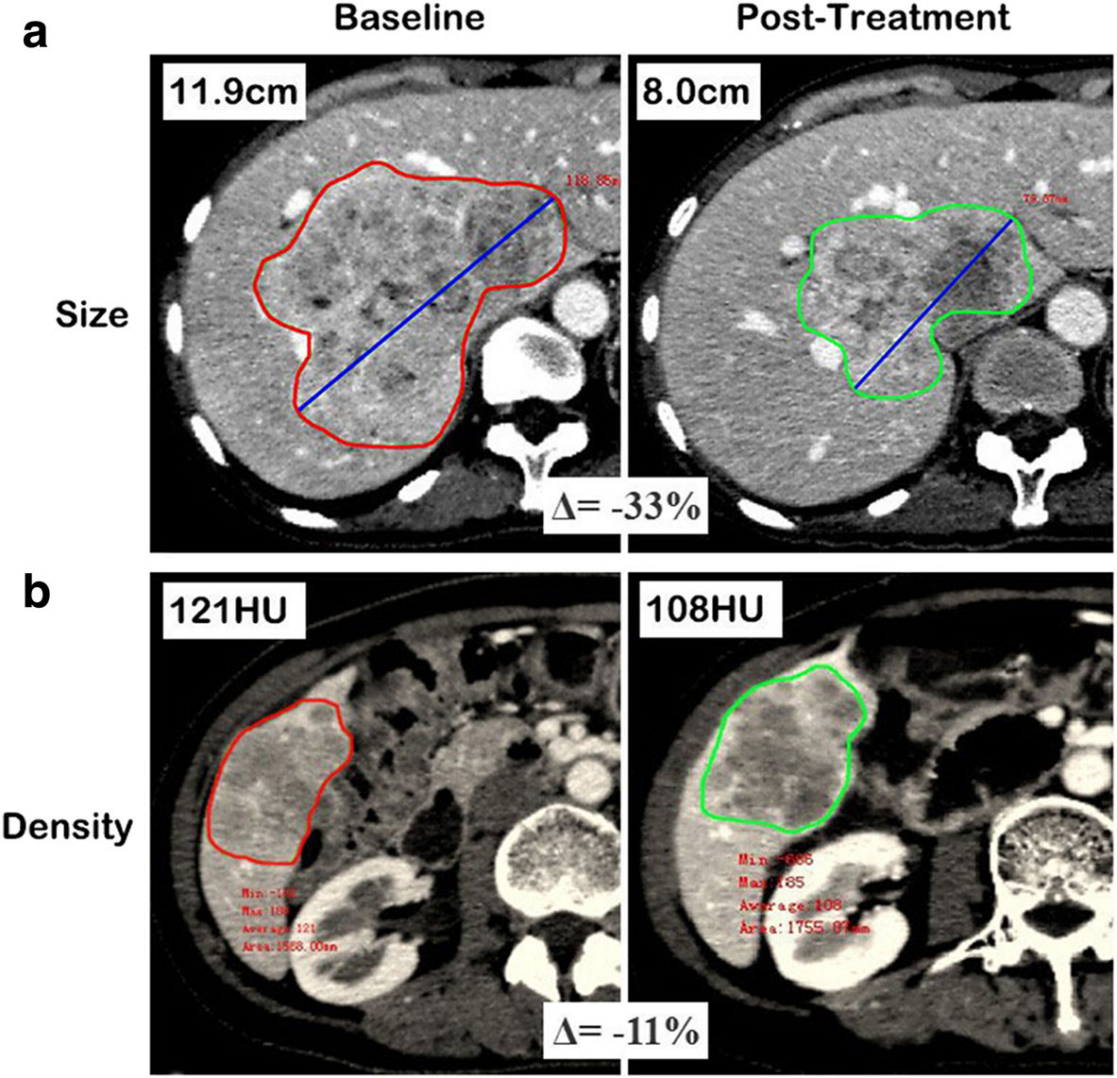
Example of evaluating percentile change in tumor size and density. Pre-treatment and post-treatment CT scans showing shrinkage of hepatic metastases from a pancreatic neuroendocrine tumor in one’s fifties, the percentile change of tumor size was (8.0–11.9)/11.9 × 100% = −33% (**a**). In another lesion in the same patient, the hepatic metastases appeared more heterogeneous, with high vascularization prior to treatment. Sunitinib induced a large area of tumor hypodensity, suggesting tumor necrosis, the percentile change of tumor attenuation was (108–121)/121 × 100% = −11% (**b**). In patients with more than one target lesion, the sum of the longest diameters/density of each target lesion in each patient was computed

The lymph nodes were measured in short axis and primary tumors and metastases were measured in long axis. Tumor size was measured using the longest cross-sectional dimension for each lesion at each time point using an advanced workstation (Vitrea 2, Toshiba Medical System, Tokyo, Japan). In patients with more than one identified target lesion, the sum of the longest diameters of each target lesion in each patient was computed. Then, the percent change in tumor size recorded between pre-treatment and the early evaluation was computed for each patient. Figure 1a displays a typical example of a tumor size evaluation.

#### Tumor density

The portal venous phase was employed for tumor density measurements. Using the advanced workstation (Vitrea2, Toshiba Medical System, Tokyo, Japan), the CT attenuation coefficient of each lesion was measured in HU by circumscribing the margin of the entire tumor in the axial plane as a region of interest. When the density evaluation was performed on more than one target lesion, the global density of the target lesions was calculated. The percent change in tumor density between pre-treatment and the early evaluation was again computed for each patient. Figure 1b displays a typical example of a tumor density evaluation.

#### TTP

TTP was defined as the time from treatment to the first evidence of tumor progression or to the last CT scan for those with no tumor progression. Tumor progression was identified on the basis of the following CT findings: the appearance of new lesions or metastasis, the appearance of new intratumoral nodules, or an increase in overall tumor size of greater than 20% [20].

### Tumor response assessment according to RECIST version 1.1 and the Choi criteria

Using the parameters mentioned above, we evaluated each individual’s responses and grouped them as complete response (CR), PR, SD or PD according to RECIST version 1.1 and the Choi criteria [20, 24] (Table 1).

**Table 1:** Comparison of RECIST version 1.1 and the Choi criteria

	RECIST version 1.1	Choi criteria
CR	Disappearance of all lesions No new lesions	Disappearance of all lesions No new lesions
PR	A decrease in size^a^ of ≥30%	A decrease in size^a^ of ≥10% or a decrease in tumor density (HU) of ≥15% in CT No new lesions No obvious progression of immeasurable disease
SD	Neither sufficient shrinkage to qualify for PR nor a sufficient increase to qualify for PD	Does not meet the criteria for CR, PR, or PD No symptomatic deterioration attributable to tumor progression
PD	An increase in size^a^ of ≥20% New lesions New intratumoral nodules or increase in the size of the existing intratumoral nodules	An increase in tumor size^a^ of ≥10% and does not meet the criteria for PR based on tumor density (HU) in CT New lesions New intratumoral nodules or an increase in the size of the existing intratumoral nodules

^a^Sum of the diameters of the target lesions defined in RECIST version 1.1 [20]

### Statistical analysis

The percent changes in tumor size and density were calculated to evaluate treatment response using both RECIST version 1.1 and the Choi criteria. The Wilcoxon rank-sum test (paired samples), the Kruskal-Wallis test (three independent samples) and the Mann–Whitney U test (two independent samples) were used for intragroup comparisons. To evaluate the ability of RECIST version 1.1 and the Choi criteria to predict prognosis, TTPs were compared between the respective groups using the Kaplan-Meier method. Kaplan-Meier curves were compared using the log-rank (Mantel-Cox) test, and all statistical analyses were performed using SPSS (Version 19.0; IBM Corp., Armonk, NY, USA). A difference with a *P* value of <0.05 was considered statistically significant.

## Results

### Study population

During the recruitment period, a total of twenty patients were treated for advanced GEP-NENs using sunitinib at our institution. A total of 18 patients met the eligibility criteria and were included in the study. The remaining 2 patients were excluded from the analysis either because of non-evaluable CT scans or evaluations performed outside the predefined interval. In total, 44 target lesions were measured in 18 patients (median, 2 lesions per patient; range, 1–4 lesions per patient). The demographic and baseline characteristics of the patients are presented in Table 2.

**Table 2:** Patients and baseline characteristics

Characteristic	
Age (yr), n (%)	
Median	47.5
Range	28–61
Sex, male/female, n (%)	8 (44.4)/10 (55.6)
Primary lesion	
Pancreas	14 (77.8)
Rectum	1 (5.6)
Unknown origin	3 (16.7)
Site of target lesions, n (%)	
Pancreas (primary tumor)	11 (25.0)
Liver	25 (56.8)
Mesenteric lymph node	1 (2.3)
Retroperitoneal lymph node	3 (6.8)
Pelvic cavity	4 (9.1)
Pathological classification, n (%)	
Grade 1	2 (11.1)
Grade 2	14 (77.8)
Grade 3	2 (11.1)
Tumor functionality, n (%)	
Nonfunctioning	16 (88.9)
Functioning	2 (11.1)
Previous treatments, n (%)	
None	11 (61.1)
Surgery	4 (22.2)
Octreotide	1 (5.6)
Transarterial chemoembolization	1 (5.6)
Transarterial chemoembolization & Octreotide	1 (5.6)
Duration of sunitinib (months)	
Median	6.8
Range	1.0–22.7
Time between initiation and first evaluation (months)	
Median	2.3
Range	1.4–3.1
Duration of follow-up (months)	
Median	17.3
Range	4.3–47.9

Among the enrolled 18 patients, five patients had dose reduced to 25 mg, the main adverse events resulted in the reduction of dosage included hand-foot syndrome and skin toxicity (*n* = 3), thrombocytopenia (*n* = 1) and neutropenia (*n* = 1). In the patient with thrombocytopenia, after dosage reduction to 25 mg/day, the patient still demonstrated persistent thrombocytopenia and subsequent treatment failure after 1.0 months. This patient demonstrated evidence of progressive disease at first follow up CT scan performed 1.4 months post treatment.

### Early assessment of changes in tumor size and density

**Fig. 2:**
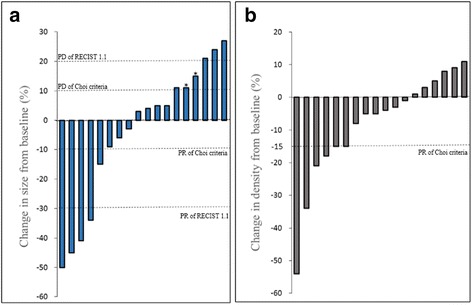
Waterfall plot of the percent change in tumor size (**a**) and density (**b**) at the first evaluation after sunitinib treatment

In the 18 evaluable patients, the total tumor size in each patient ranged from 2.6 to 28.5 cm (median, 13.7 cm) before treatment and from 3.2 to 30.0 cm (median, 10.9 cm) at the first evaluation after treatment. Tumor density ranged from 63 to 478 HU (median, 179 HU) before treatment and from 68 to 453 HU (median, 144 HU) at the first evaluation after treatment. No significant difference (*Z* = −0.348,*P* = 0.727) in tumor size was observed between the baseline and the first evaluation. However, a significant decrease (*Z* = −2.309,*P* = 0.021) in tumor density was detected among the evaluable lesions. Figure 2 shows the percent change in tumor size and density measured via CT scans at baseline and at the first evaluation after treatment with sunitinib for all evaluable patients.

### Early response assessed using RECIST 1.1 and the Choi criteria

**Fig. 3:**
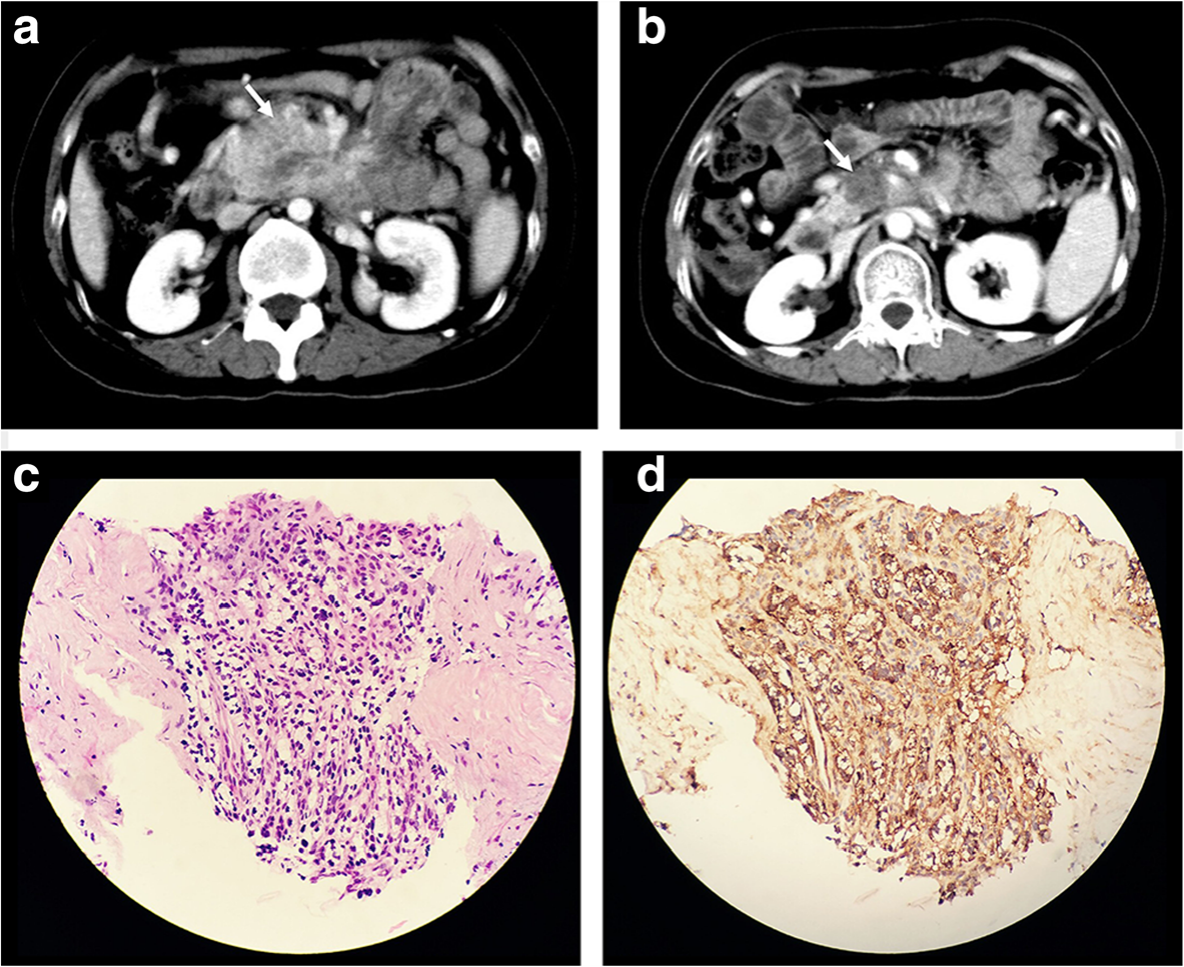
A primary pancreatic neuroendocrine tumor with multiple hepatic metastases (G2) in one’s fourties. (**a**) Pre-treatment CT scan showing a large mass in the pancreatic body with a heterogeneous, hyperdense tumor (white arrow, size: 5.5 cm, density: 91 HU). (**b**) CT scan obtained 2.8 months after treatment of sunitinib showing that the lesion had become significantly smaller in size and more hypodense (white arrow, size: 2.5 cm, density: 44 HU). The percent change in tumor size and density was 55% and 52%, respectively, which was classified as PR by both the Choi criteria and RECIST. Samples obtained through endoscopic ultrasound-guided fine needle tissue acquisition before treatment showing a large trabecular structure, moderate cell atypia (**c**, original magnification, ×200, hematoxylin-eosin staining) and intense immunoreactivity for VEGFR2 (**d**, original magnification, ×200, IHC staining)

**Fig. 4:**
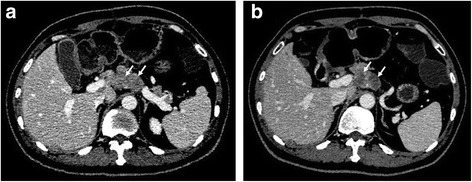
A pancreatic neuroendocrine tumor (G2) with retroperitoneal lymph node metastases in one’s fifties. The pre-treatment CT scan showed (**a**) retroperitoneal fusion nodules with a relatively low density (white arrows, size: 2.0 cm, density: 82 HU) in front of the abdominal artery. (**b**) The nodules exhibited a slight reduction in size and an obvious reduction in density (white arrows, size: 1.9 cm, density: 56 HU) at the first evaluation after treatment with sunitinib. The percent change in tumor size and density was 5.0% and 31.7%, respectively. This patient was classified as PR according to the Choi criteria but as SD based on RECIST

Tumor response to sunitinib was evaluated using RECIST 1.1 and the Choi criteria. Among the 18 patients evaluated in this trial, no patient demonstrated CR after treatment with sunitinib, 4 patients (22.2%) demonstrated PR, while 9 patients (50.0%) demonstrated SD, and 5 patients (27.8%) demonstrated PD according to RECIST 1.1. Based on the Choi criteria, 8 (44.4%) of 18 evaluable patients demonstrated PR (Figs. 3 and 4), 4 patients (22.2%) demonstrated SD and 6 patients (33.3%) demonstrated PD. Among the patients classified as showing PD by RECIST 1.1, 2 developed new lesions, and the other 3 showed an increase in tumor size of greater than 20% according to the various criteria.

The median changes in tumor size and density in the PR, SD and PD groups according to both RECIST 1.1 and the Choi criteria are shown in Table 3 (placed at the end of the document text file). The changes in tumor size were significantly different in the three groups according to both RECIST 1.1 and the Choi criteria (*P* < 0.05). The differences observed in the change of tumor density were statistically significant according to the Choi criteria (*P* = 0.042), but not according to RECIST 1.1 (*P* = 0.119).

**Table 3:** Median changes in tumor size and density according to RECIST 1.1 and the Choi criteria in evaluable patients (%)

Parameter	RECIST 1.1		Choi criteria	
	Size	Density	Size	Density
PR	−43.4	−17.8	−24.9	−16.8
SD	3.5	−5.8	3.8	−5.0
PD	21.0	1.2	18.2	3.2
Z value	13.888	4.256	13.263	6.352
*P* value	0.001	0.119	0.001	0.042

### Assessment of TTP according to RECIST 1.1 and the Choi criteria

**Fig. 5:**
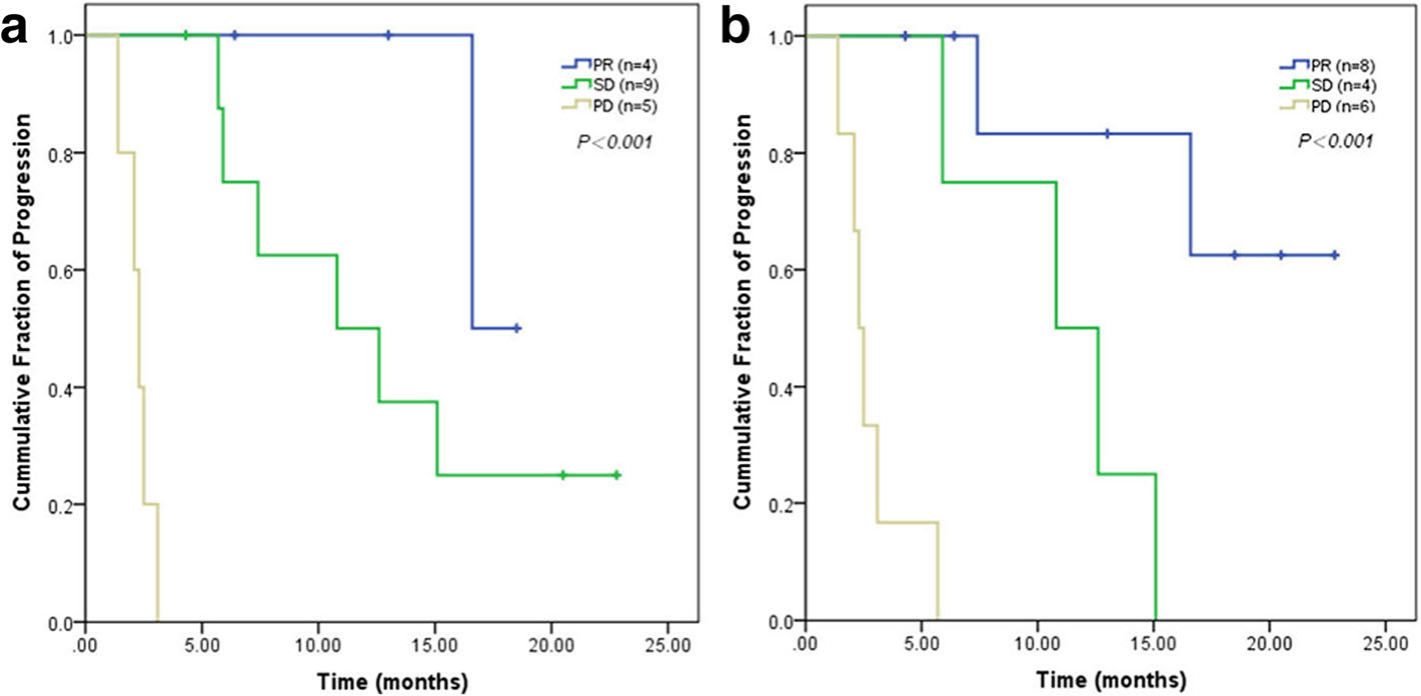
Kaplan-Meier analyses of the TTP in the PR, SD and PD groups, as classified according to RECIST 1.1 (**a**) and the Choi criteria (**b**)

During the trial, we monitored tumor progression was observed in twelve patients. The remaining 6 patients (33.3%) exhibited no evidence of tumor progression. The median TTP of these18 patients was 10.8 months. A significant difference in TTP was observed in the PR, SD and PD groups when using both sets of criteria (*P <* 0.001, Fig. 5). Based on the RECIST 1.1, the median TTP of PR, SD and PD were 16.6 months, 10.8 months and 2.3 months, respectively. According the Choi criteria, the median TTP of PR, SD and PD group were not reached, 10.8 months and 2.3 months, respectively. The results of secondary analyses for TTP showed that according to RECIST 1.1, there was a significant difference in TTP between the PR and PD groups (*P* = 0.007) and between the SD and PD groups (*P <* 0.001), but there was no significant difference between the PR and SD groups (*P* = 0.131). Based on the Choi criteria, the TTP of the PR group was significantly longer than those observed in the SD (*P* = 0.026) and PD groups (*P <* 0.001), and the TTP of the SD group was significantly longer than that in the PD group (*P* = 0.006). Table 4 showed the percentile change of tumor size, tumor density, early response to Sunitinib by RECIST 1.1 and Choi criteria, and TTP of each patient.

**Table 4:** Early evaluation of tumor response to sunitinib by RECIST 1.1 and Choi criteria

No. of patient	Change in tumor size (%)	Change in tumor density (%)	RECIST 1.1 response	Choi response	TTP (m)
1	−34	−15	PR	PR	16.6
2	−42	−21	PR	PR	6.4
3	−45	11	PR	PR	18.5
4	−50	−54	PR	PR	13.0
5	−15	3	SD	PR	7.4
6	5	−15	SD	PR	22.8
7	−6	−18	SD	PR	20.5
8	−9	−34	SD	PR	2.4
9	−3	−8	SD	SD	10.8
10	5	−5	SD	SD	15.1
11	4	−3	SD	SD	5.9
12	3	−5	SD	SD	12.6
13	11	5	SD	PD	5.7
14	11	−1	PD^a^	PD	1.4
15	27	9	PD	PD	3.1
16	15	−4	PD^a^	PD	2.3
17	24	1	PD	PD	2.1
18	21	8	PD	PD	2.5

^a^The Progressive Disease of RECIST version 1.1 was the result of new lesions at the first follow up by CT scan, rather than the increase of tumor size

## Discussion

The response of solid tumors to treatment has traditionally been evaluated using RECIST 1.1. According to RECIST 1.1, PR corresponds to a >30% decrease in the sum of the maximum diameters of the target lesions, which is the current standard for assessing the response of solid tumors to anticancer therapy [20]. However, for targeted therapies, which generally reduce tumor vascularization, subsequently inducing necrosis and cystic degeneration, the change in tumor density can also be measured from clinical images as a parameter for evaluating the response to targeted therapies [22]. Nevertheless, changes in tumor density may have no major effect on tumor size during targeted therapy and are frequently categorized as SD when using RECIST. Therefore, the application of RECIST which fails to identify clinical response to targeted therapies in this group of patients carries the risk of prematurely terminating the use of active targeted drugs. The Choi criteria, which incorporate changes in both tumor density and size measured via CT, have been demonstrated to be more sensitive and accurate than RECIST for predicting imatinib efficacy in GISTs [23, 24]. Faivre S, et al. have reported the use of the Choi criteria as an alternative to RECIST for evaluating the effects of sunitinib in patients with advanced pancreatic NENs only, however, it was not used to evaluate midgut NENs in that study [25, 26]. The present study was conducted to compare the two sets of criteria based on the data obtained from a homogenous group of patients with advanced GEP-NENs treated with sunitinib. A comparison of the Choi criteria with RECIST demonstrated that the Choi criteria were more precise in assessing the early response of GEP-NENs to sunitinib.

In the present study, both tumor size and density were decreased after treatment with sunitinib. No statistically significant difference was observed in tumor size before and after treatment (Z = −0.348, *P* = 0.727), whereas the difference in tumor density before and after treatment was statistically significant (Z = −2.309, *P* = 0.021). Similar results have been reported previously for HCC [29, 30]. Faivre et al. observed the striking appearance of large areas of tumor hypodensity during treatment with sunitinib in HCC, despite limited changes in tumor size [30]. Zhu and colleagues reported that sunitinib significantly reduced intratumoral vascularization, leading to significant changes in the transfer constant K_trans_, a surrogate endpoint for vessel leakage [30, 31]. These features are believed to reflect the inhibitory effects of sunitinib on vascular endothelial cell VEGFR expression and on tumor pericyte PDGFR expression, resulting in disrupted, congested, tortuous, and leaking tumor vessels associated with necrotic areas in the tumor, rather than significant shrinkage of tumor cells [18], which may indicate that a reduction in tumor density is a more sensitive parameter than tumor size in evaluating the early responses to sunitinib treatment.

According to both RECIST 1.1 and the Choi criteria, the differences in the change in tumor size observed in the PR, SD and PD groups were statistically significant (*P* < 0.005). The differences in the change in tumor density among the three groups based on RECIST 1.1 were not statistically significant (*P* = 0.119), but a significant difference in the change in tumor density was observed according to the Choi criteria (*P* = 0.042). These results indicate in addition to RECIST 1.1 where treatment reponse is based solely on change in tumor size, the Choi criteria also reflect differences in histological composition during the treatment of GEP-NENs with sunitinib, which clearly originates from the definitions of RECIST and the Choi criteria [20, 24].

The Choi response criteria, which incorporate changes in both tumor density and size observed in contrast-enhanced CT scans, classified twice as many of the 18 GEP-NEN patients as PR (44.4%) compared with RECIST 1.1 (22.2%). Moreover, the TTP was significantly longer in patients classified as PR according to the Choi criteria than in those classified as SD (*P* = 0.026) and PD (*P* < 0.001). According to RECIST 1.1, statistically significant differences in TTP were observed between the PR and PD groups (*P* = 0.007) and between the SD and PD groups (*P* < 0.001). However, using RECIST 1.1, TTP was not significantly longer in patients classified as PR than in those classified as SD (*P* = 0.131). These results suggested that patients with advanced GEP-NENs who were categorized into the PR group according to the Choi criteria during the early tumor evaluation experienced better outcomes than those in the SD group. This finding is consistent with the ones suggested Benjamin and Choi et al., who also raised this issue regarding the inadequacy of RECIST in identifying responding tumors. This limitation of RECIST may be due to the way sunitinib functions as an antiangiogenic agent [23, 24].

Unlike RECIST 1.1, the Choi criteria do not provide clear definitions for evaluating TTP in patients. Therefore, in this study, we were unable to define TTP according to the Choi criteria. At the end of this trial, 12 patients demonstrated evidence of tumor progression, and the TTP could not be established in 6 patients, as illustrated in the results. It is likely that RECIST 1.1 and the Choi criteria would converge in defining similar rates of progression. Furthermore, in the work reported by Choi and colleagues, who compared the Choi criteria and RECIST 1.1 in evaluating the efficacy of imatinib against metastatic GISTs, the TTP was defined as the same rate of disease progression as in RECIST 1.1 in both groups [24].

Another limitation of this trial include small sample size which may result in certain degree of research bias. However, this study remains significant as it may help to identify the need to cooperate a better criteria for clinical evaluation of tumor response.

## Conclusion

In conclusion, the Choi criteria appears to be more appropriate than RECIST 1.1 in identifying clinical responses as longer TTP observed in Choi represents better efficacy of sunitinib in advanced GEP-NENs. The limitations of a small sample size and intermediate follow-up period may result in certain degree of research bias. Future studies with large sample sizes and long enough follow-up times should be conducted to further explore the most appropriate criteria in evaluating the tumor response to sunitinib treatment.

## Abbreviations

CR: Complete response
CT: Computed tomography
GEP-NENs: Gastroenteropancreatic neuroendocrine neoplasms
GISTs: Gastrointestinal stromal tumors
HCC: Hepatocellular carcinoma
HU: Hounsfield units
IHC: Immunohistochemical
PD: progressive disease
PDGFRs: Platelet-derived growth factor receptors
PR: Partial response
RECIST: Response evaluation criteria in solid tumors
SD: Stable disease
TTP: Time to tumor progression
VEGFRs: Vascular endothelial growth factor receptors.
